# Chemodiversity of Soil Dissolved Organic Matter and Its Association With Soil Microbial Communities Along a Chronosequence of Chinese Fir Monoculture Plantations

**DOI:** 10.3389/fmicb.2021.729344

**Published:** 2021-10-21

**Authors:** Ying Li, Kate Heal, Shuzhen Wang, Sheng Cao, Chuifan Zhou

**Affiliations:** ^1^University Engineering Research Center of Sustainable Plantation Management, Forestry College, Fujian Agriculture and Forestry University, Fuzhou, China; ^2^Institute of Quality Standards and Testing Technology for Agro-Products, Fujian Academy of Agricultural Sciences, Fuzhou, China; ^3^School of GeoSciences, The University of Edinburgh, Edinburgh, United Kingdom

**Keywords:** DOM, soil quality, bacteria, fungi, Chinese fir, chemodiversity

## Abstract

The total dissolved organic matter (DOM) content of soil changes after vegetation transformation, but the diversity of the underlying chemical composition has not been explored in detail. Characterizing the molecular diversity of DOM and its fate enables a better understanding of the soil quality of monoculture forest plantations. This study characterized the chemodiversity of soil DOM, assessed the variation of the soil microbial community composition, and identified specific linkages between DOM molecules and microbial community composition in soil samples from a 100-year chronosequence of Chinese fir monoculture plantations. With increasing plantation age, soil total carbon and dissolved organic carbon first decreased and then increased, while soil nutrients, such as available potassium and phosphorus and total nitrogen, potassium, and phosphorus, increased significantly. Lignin/carboxylic-rich alicyclic molecule (CRAM)-like structures accounted for the largest proportion of DOM, while aliphatic/proteins and carbohydrates showed a decreasing trend along the chronosequence. DOM high in *H*/*C* (such as lipids and aliphatic/proteins) degraded preferentially, while low-*H*/*C* DOM (such as lignin/CRAM-like structures and tannins) showed recalcitrance during stand development. Soil bacterial richness and diversity increased significantly as stand age increased, while soil fungal diversity tended to increase during early stand development and then decrease. The soil microbial community had a complex connectivity and strong interaction with DOM during stand development. Most bacterial phyla, such as *Acidobacteria*, *Chloroflexi*, and *Firmicutes*, were very significantly and positively correlated with DOM molecules. However, *Verrucomicrobia* and almost all fungi, such as *Basidiomycota* and *Ascomycota*, were significantly negatively correlated with DOM molecules. Overall, the community of soil microorganisms interacted closely with the compositional variability of DOM in the monoculture plantations investigated, both by producing and consuming DOM. This suggests that DOM is not intrinsically recalcitrant but instead persists in soils as a result of simultaneous consumption, transformation, and formation by soil microorganisms with extended stand ages of Chinese fir plantations.

## Introduction

Plantation forests provide a variety of goods and services, which alleviate the pressure on natural forest resources (Tuomela et al., 2000; Peltoniemi et al., 2004; García-Palacios et al., 2016; Liu et al., 2018; Silva et al., 2019). The global area of planted forests has gradually increased over the past 20 years, and with an average annual increase of 4.3 × 10^4^ km^2^, it currently accounts for 6–7% of forests globally (Zhijun et al., 2018). However, due to the single structure of forest stands, the lack of species diversity, and removal of nutrients in harvested biomass, plantation forests are limited by the availability of soil nutrients (Suleiman et al., 2019; Zhang M. et al., 2019).

Studying the dynamic changes of forest soil quality in response to forest age can help to clarify the interaction (and its underlying mechanisms) between aboveground vegetation and soil. Furthermore, the results provide a scientific basis for sustainable forest management and the improvement of soil fertility (Zhang M. et al., 2019). Plant species composition and canopy closure affect the forest internal environment, which inevitably influences the soil environment with increasing stand age (Chen et al., 2019). Furthermore, as plants grow, the balance between the soil nutrient supply and nutrient demand for tree growth also changes.

It has been shown that soil nutrient concentrations in forest plantations increase with increasing forest age (Liu J. et al., 2016) in stands of *Pinus massoniana*. Soil total phosphorus (TP), available phosphorus (AP), total nitrogen (TN), and available nitrogen (AN) concentrations, as well as the soil *N*/*P* ratio, increased with stand ages (>30 years). Similarly, the carbon stock of the organic soil layer was augmented by an average of 4.7 g m^–2^ a^–1^ with increasing stand age of *Pinus sylvestris* up to 125 years (Peltoniemi et al., 2004). Contrastingly, it has been suggested that soil nutrients decrease gradually with forest age. The nutrient content of middle-aged *Nothofagus antarctica* forests (21–110 years) was reported to have decreased significantly compared to young (5–20 years) forests (Peri et al., 2006). Wu et al. (2015) found a decline in soil nutrient concentrations with stand age from 3 to 25 years for *Pinus elliottii* plantations in subtropical China, which further indicates that soil nutrients may be deficient with increasing stand ages in a monoculture forest plantation (Peri et al., 2006). These conflicting findings clearly demonstrate the need for mechanistic understanding of how soil ecosystems, including dissolved organic matter and microorganisms, change with stand development in monoculture plantations (Guillaume et al., 2016; Liu S. et al., 2016; Awale et al., 2017).

Dissolved organic matter (DOM), while only accounting for a small fraction of organic matter, is the most active component of the soil organic matter pool. It plays an important role in the regulation of mineral weathering; cationic leaching; metal dissolution; microbial activity; and other soil physical, chemical, and biological processes (Peri et al., 2006). In addition, DOM could provide soluble organic matters that give assistance to heterotrophic microbial communities (Li et al., 2019). Fourier-transform ion cyclotron resonance mass spectrometry (FT-ICR MS) reveals the detailed molecular formulae and basic structural features of DOM, which enable characterization of its chemodiversity (Kellerman et al., 2014). Furthermore, variations of DOM composition can have profound effects on microbial behavior, reproduction, and survival and *vice versa*. The association between individual DOM molecules and soil microbes underpins the DOM cycle. Understanding of the interaction between soil microbes and DOM molecules, which supports soil microbial ecology, carbon cycling, and the provision of ecological services in forest plantations, is fundamental for the sustainable use of forest plantation soils. However, the DOM chemodiversity and the relationships between DOM and microbial structure and composition in monoculture forest plantations have not been reported to date.

*Cunninghamia lanceolata* (lamb.) Hook. (Chinese fir) is one of the most important timber species in southern China due to its excellent timber, rapid growth, and high yield. It plays an essential role in forestry production in China and is currently cultivated across an area of 12 million ha in China (Zhou et al., 2019a). However, such an increase has arisen from the conversion of natural forests to either Chinese fir monoculture plantations or mixed plantations of Chinese fir and Moso bamboo (*Phyllostachys edulis*) that now cover 60–80% of the timber plantation area in southeast China (Selvaraj et al., 2017).

The main purpose of this study was to investigate the DOM molecular composition and the microbial community composition of soil from Chinese fir plantations with different stand ages up to 100 years. The chemodiversity of forest soil DOM was characterized, and its correlation with soil bacterial and fungal community was analyzed. The hypotheses were as follows: (1) soil DOM chemodiversity increases with increased stand ages of Chinese fir plantations, (2) the microbial community richness changes due to the transformation of available organic carbon content, and (3) the soil DOM chemodiversity is positively or negatively correlated with bacterial and fungal community composition across a chronosequence that spanned 100 years. This study increases understanding of carbon sink management in monoculture forest soils and provides new insight into the relationship between soil microbes and DOM.

## Materials and Methods

### Study Area and Sample Collection

The study area is a Chinese fir monoculture plantation, which lies in a small watershed in Wangtai Town (26° 38′-26° 42′ N, 117° 54′-117° 57′ E), Nanping City, Fujian Province, China. This area has a subtropical monsoon climate, with the following mean annual characteristics reported for 1981–2010 (Guo et al., 2014). Annual temperature of 19.3°C and a relative humidity of 83%. The mean annual precipitation is 1,699 mm, mostly occurring from March to August, and the mean annual evapotranspiration is 1,413 mm. The elevation of the study area ranges from 150 to 250 m above sea level, and the slope angle is ∼30°. The soil is red earth derived from granite, which is equivalent to the Hapludult soil group in the United States Department of Agriculture (USDA) soil taxonomy (Selvalakshmi et al., 2018). The soil texture varies from sandy clay to clay loam. The soil profile is well developed with charcoal deposition in the organic layer, due to slash burning and cutting of the previous natural forest cover to establish the Chinese fir plantations. The Chinese fir plantations in the study area were tended twice a year for the first 5 years after planting of seedlings, with few management measures applied subsequently (Selvalakshmi et al., 2018).

### Soil Sampling and Preparation

**FIGURE 1:**
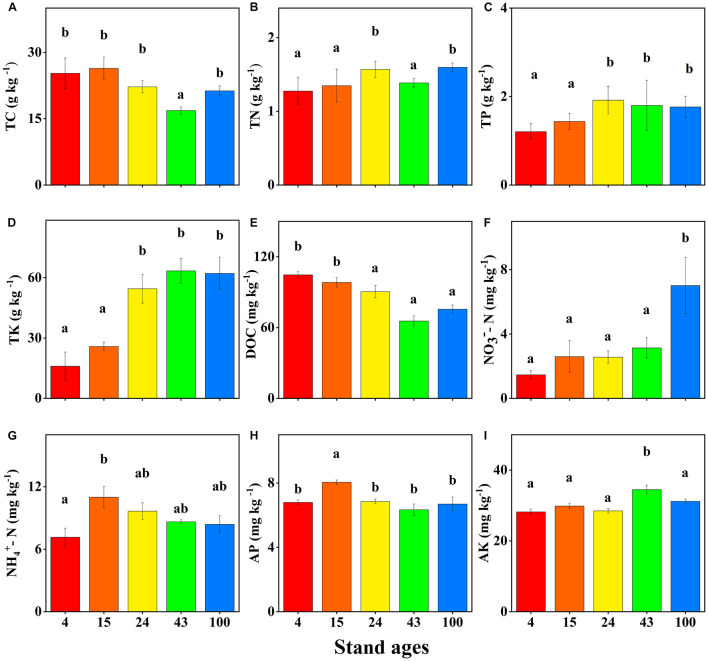
Soil nutrient concentrations in different stand ages (years) of Chinese fir plantations (mean ± SE, *n* = 3). Total carbon (TC) **(A)**, total nitrogen (TN) **(B)**, total phosphorus (TP) **(C)**, total potassium (TK) **(D)**, dissolved organic carbon (DOC) **(E)**, nitrate-nitrogen (NO_3_^–^-N) **(F)**, ammonium-nitrogen (NH_4_^+^-N) **(G)**, available phosphorus (AP) **(H)**, and available potassium (AK) **(I)**. DOC, NO_3_^–^-N, and NH_4_^+^-N concentrations are expressed as milligrams per kilogram fresh weight. Bars with different letter(s) are significantly different between stand ages (*p* < 0.05).

In 2018, three replicate stands were selected in Chinese fir plantations of five different ages (4, 15, 24, 43, and 100 years, the soil background information shown in Figure 1 and images shown in Supplementary Figure 1) for the study (Cao et al., 2021). Further characteristics of each stand age are given in Supplementary Table 1. Three replicates within each stand age were carefully selected to be as similar as possible to enable comparison of soil quality. The Chinese fir plantation stands were also selected as close to each other as possible to minimize variation among sites, which were no further than 4 km apart. The sampling was conducted using the same method as the earlier study by Selvaraj et al. (2017). In each stand, six plots (20 m × 20 m) were selected in an S-shape at mid-slope positions to minimize the effect of slope on soil characteristics; soil samples were collected at 0–20 cm depth. The soils from the six plots were thoroughly mixed to form a single composite sample (∼2 kg) for each replicate stand, which were placed in an air-tight bag in a cool box (4°C) for transport to the laboratory.

In the laboratory, seeds, roots, gravel, and other materials were removed from all the soil samples by hand. Part of the soil samples was air-dried and passed through a 2-mm sieve for determination of pH, dissolved organic carbon (DOC), and AP, and through a 0.149-mm sieve for TP, TN, total carbon (TC), and total potassium (TK) analysis. Fresh soil samples were passed through a 2-mm sieve for later determination of concentrations of available ammonium-nitrogen (NH_4_^+^-N), nitrate-nitrogen (NO_3_^–^-N), and DOC and characterization of soil DOM composition and the soil microbial community (Lu, 1999).

### Soil Chemical Analysis

Three soil samples of each stand age were analyzed. Soil pH was determined by potentiometry (Ohaus ST3100, NJ, United States) in a suspension of 10 g of air-dried soil (<2 mm) in 25 ml of double-deionized water, after stirring intermittently for 30 min (Minghe and Ritchie, 1999). Available NO_3_^–^ and NH_4_^+^ were determined in 3 g fresh soil extracted with 30 ml of 2 mol l^–1^ KCl solution, then shaken in a shaker (SPH-2102C, Shiping, China) for 30 min, and centrifuged for 5 min at 2,800 × *g* (5810 R, Eppendorf, Germany). The supernatant was filtered using a 0.45-μm PES membrane filter (Jinteng, Tianjin Jinteng Experiment Equipment Co., Ltd., China), then the concentrations of nitrate and ammonium were determined by a continuous flow analyzer (SkALAR SAN++, Netherlands) (Jones and Willett, 2006). The instrument was calibrated with five standards and a blank (Millipore Water), and a standard was analyzed for quality control every 25 samples. Nitrogen concentrations in a blank extract in KCl solution only with no soil present were subtracted from the soil sample results. The results of nitrate-nitrogen (NO_3_^–^-N) content and ammonium-nitrogen (NH_4_^+^-N) are shown on a fresh weight basis.

Soil TC and TN were measured by dry combustion of 0.1 g subsamples in an Automatic Trace Element Analyzer (Vario MAX Elementar, Germany) (Jones and Willett, 2006). Soil DOC was extracted by adding 50 ml of 2 M l^–1^ KCl to a tube, which contained 5 g fresh soil sample, and then conducting shaking, centrifugation, and filtration as for the available N extractions. Finally, DOC concentration was measured using a total organic carbon analyzer (TOC-L CPH Analyzer Shimadzu, Japan) (Jones and Willett, 2006).

The soil TP and TK concentrations were determined by ICP-OES (PerkinElmer, United States) on 0.15 g sub-samples digested with 10 ml of 14.5 M l^–1^ nitric acid (HNO_3_) at 120°C for 24 h. The instrument was calibrated (five standards) and a blank (Millipore Water), and standards were analyzed for quality control every 25 samples (Selvalakshmi et al., 2018). Available P was determined in 5.0 g air-dried soil extracted with a 50-ml mixture of 0.025 M l^–1^ HCl solution and 0.03 M l^–1^ of NH_4_F, agitating them on an oscillating shaker (SPH-2102C, Shiping, China) for 5 min, before filtration through P-free filter paper to extract AP (Whatman, China) (Lu, 1999; Liu et al., 2017). Phosphorus concentration in the filtrates was measured using the molybdenum blue method with an ultraviolet-visible spectrophotometer (Puxi/T6, China) at 700 nm (Lu, 1999). Available *K* was determined in 5.0 g air-dried soil (passed through a 2-mm mesh) samples extracted with 50 ml of 1 M l^–1^ CH_3_COONH_4_ (pH 7.0), shaken for 30 min (SPH-2102C, Shiping, China) (Lu, 1999; Zhang H.Q. et al., 2019), and then filtered through a filter paper (Whatman, China), before measurement with an atomic absorption spectrophotometer (TAS-990, Puxi, China). The instruments used to measure AP and AK concentrations were calibrated with five standards and a blank (Millipore Water).

### Dissolved Organic Matter Electrospray Ionization Fourier-Transform Ion Cyclotron Resonance Mass Spectrometry Analysis

To understand the differences in DOM composition of soils under Chinese fir plantations of different ages, the molecular characteristics were determined of DOM extracted by solid-phase extraction and analyzed by FT-ICR MS (Bruker-SolariX, United States). Briefly, 12 g of a composite soil sample from stands of each plantation age was taken from a cryogenic refrigerator and placed in a centrifuge tube with 60 ml of Milli-Q water (18 MΩ). The tube was shaken on a reciprocating shaker (170 rpm) for 8 h (25°C constant temperature), then centrifuged at 2,800 × *g* for 10 min, and the supernatant was filtered (0.45 μm) with a suction bottle (Li et al., 2019). Samples were flushed with mass spectrometry-grade pure methanol, the solid-phase extraction box was activated (Bond Elut PPL, 6 ml, 500 mg, Agilent Technology), and then DOM samples were flushed and acidified in filter cartridges with acidified (pH 2) Milli-Q water. After complete drying of the cartridge with super pure N_2_ gas (99.999% purity by volume), DOM was eluted from the cartridge with methanol (5 ml) to give about 20 mg l^–1^ DOC (Li et al., 2019). The molecular composition of the extracted soil DOM samples was analyzed by electrospray ionization (ESI) FT-ICR MS equipped with 9.4-T superconducting magnets. The ESI charge can preferentially ionize polar heteroatom compounds (e.g., nitrogen and sulfur) in the presence of hydrocarbons. Here, negative ESI mode was used as it favors detection of molecules with acidic functional groups that deprotonate, such as carboxylic acids, and thus is the mode commonly used in studies of natural organic matter (e.g., Yuan et al., 2017; Li et al., 2018). The following operating conditions for negative-ion formation were used: 4 kV spray shielding voltage, 4.5 kV capillary column voltage, and 320 V capillary end voltage. The mass range was set at 200–800 *m*/*z* (Thieme et al., 2019).

Electrospray Ionization FT-ICR MS provides high-accuracy data on the masses of compounds, and it is this that enables the composition of molecules to be determined. An in-house mass reference list was used for internal calibration. According to the different cyclotron periods of ions with different *m*/*z* ratio, the frequency of current signal is transformed into the *m*/*z* ratio of ions by Fourier transform, and the intensity of signal reflects the abundance of ions (but not its concentration). Based on the *m*/*z* ratio range inputted, a dictionary of possible molecular formulae is generated. Detailed information on mass calibration, data acquisition, and processing of FT-ICR MS have been reported previously (e.g., Yuan et al., 2017).

### Soil Microbial Community Analysis

The TruSeq^®^ DNA PCR-Free Sample Preparation Kit (Illumina, United States) was used to generate sequencing libraries (Zhang et al., 2018). On an Illumina Hiseq 2500 sequencing platform, 16S rRNA and ITS-1 sequence analysis was conducted. A DNA Kit (Omega Bio-tek, Norcross, GA, United States) was used to extract the total DNA genome of soil samples (three replicates for each stand age). For quality detection, 1% agarose gel electrophoresis detection and spectrophotometry (260 nm/280 nm optical density ratio) were used. The obtained DNA was amplified using primers 338F (5′-ACTCCTACGGGAGGCAGCAG-3′) and 806R (5′-GGACTACNNGGG TATCTAAT-3′), which target the V3–V4 16s rRNA region (Chen et al., 2016). The unique multiplex identifiers per sample of fungi are ITS1 (5′-CTTGGTCATTTAGAGGAAGTAA-3′) and ITS2 (5′-TGCGTTCTTCATCGATGC-3′), and three replicates were conducted per sample. Equal amounts of purified amplification products were mixed and tested after the sequence library was constructed. The original sequence was uploaded to the NCBI SRA database. The raw sequenced reads were merged (Flash v1.2.7), quality controlled, and filtered (Trimmomatic v0.33) (Zhang et al., 2018; Madaha et al., 2020). Chimeric sequences were removed with the UCHIME (v 4.2) method (Edgar, 2016) in mothur software (Edgar et al., 2011).

### Data Analysis

Statistical analyses were conducted using SPSS v.22. One-way ANOVA and comparison of means with LSD’s honestly significant difference *post hoc* test (*p* < 0.05) were conducted to identify any significant differences in soil chemical properties and microbial community diversity among the stand ages. After the characterization of soil DOM by FT-ICR MS, compound groups were delineated by the following parameters: *H*/*C* and *O*/*C* ratios and the double bond equivalence (DBE) value, which reflects the degree of unsaturation of the DOM molecule (Melendez-Perez et al., 2016). The intensity-weighted averaged values of the *H*/*C* ratio, *O*/*C* ratio, DBE, and *m*/*z* of DOM molecules were calculated as follows:


(1) $$X/C_{\mathrm{wa}}=\sum \left(\frac{{\text{X}}_{\text{i}}}{{\text{C}}_{\text{i}}}\times {\text{M}}_{\text{i}}\right)$$

(2) $${\text{DBE}}_{i}=1-{\text{C}}_{i}-\frac{{\text{H}}_{\text{i}}-{\text{N}}_{\text{i}}}{2}$$

(3) $${\text{DBE}}_{\mathrm{wa}}=\sum \left({\text{DBE}}_{\text{i}}\times {\text{M}}_{\text{i}}\right)$$

(4) $$m/z_{\mathrm{wa}}=\sum \left(\frac{m}{z_{\text{i}}}\times {\text{M}}_{\text{i}}\right)$$

(5) $${\text{M}}_{\text{i}}=\frac{{\text{I}}_{\text{i}}}{\sum {\text{I}}_{\text{i}}}$$

where *I*_i_ is the intensity value of the DOM formula; *C*_i_ is the number of C atoms; *X*_i_ is the number of H and O atoms; *M* is the molecular mass of each molecular formula; and *m*/*z* is the relative molecular weight of DOM formula (Shang et al., 2019).

In addition, the identified DOM formulae were visualized with a van Krevelen diagram and were grouped into seven distinct biochemical classes according to previous literature (Li et al., 2018). We applied the Shannon index and the Pielou index to assess quantitatively the chemodiversity of DOM molecules by analogy with the diversity calculation methods in classical ecology. The molecular formulae of compounds were treated as species, and the observed intensity was used in place of the relative abundance of species. The Shannon index and the number of detected DOM molecular formulae were used to calculate the evenness of DOM compounds (Kellerman et al., 2014; Noriega-Ortega et al., 2019).

The quantity of soil microbial community operational taxonomic units (OTUs) determined by high-throughput sequencing was converted to their relative abundance. Mothur (v.1.30) software was used to evaluate alpha diversity indices of subsamples (Ma et al., 2019). Alpha diversity indicators of soil bacteria and fungi, including the number of observed OTUs, Chao1 richness, Simpson’s diversity index, Shannon’s diversity index, and Faith’s phylogenetic diversity (PD), were calculated for each site (Nie et al., 2018) and compared by single-factor ANOVA. Principal component analysis (PCA) and mapping of the soil bacterial and fungal communities for the different Chinese fir stand ages were conducted in R (Wang et al., 2012). Redundancy analysis using Canoco v.4.5 was used to identify associations between soil microbial communities and soil nutrients and DOM compounds. A partial Mantel test was conducted using the Tutools platform^1^ to determine the soil environmental driving factors in bacterial and fungal communities by correlating bacterial and fungal communities (number of OTUs) with soil chemical properties and DOM composition. Pairwise correlations were calculated to determine any significant connection (*p* < 0.01) between specific DOM molecules and microbial community OTUs using the Pearson correlation coefficient. These values were then visualized in a network (Cytoscape software v.3.5.1, United States), prioritized according to the correlation value to decrease network complexity and thus allow identification of key linkages between DOM molecular and soil microbial community compositions.

## Results and Discussion

### Soil Nutrient Content of Chinese Fir Plantations of Different Ages

The chemical properties of soil differed significantly between different ages of Chinese fir plantations. Figure 1 shows that there is a decreasing trend of soil TC concentration as forest age increases, but it was only significantly lower in the 43-year-old stand. This trend may be the result of burning, which leads to the highest TC concentration in the young plantations (Zhang Y. et al., 2016; Selvalakshmi et al., 2018). Soil DOC concentrations decreased significantly with Chinese fir stand development, which is consistent with Zhang Y. et al. (2016), from at least 24-year-old stands onwards. The higher soil DOC concentration in the young plantations may be due to the burning of native vegetation to establish the Chinese fir plantations, since a number of studies in other ecosystems have reported increased soil DOC concentrations after burning. For example, Clay et al. (2009) found that in the weeks following a controlled management burn, there were peaks in DOC concentration in the soil water of burnt plots compared to unburnt controls. Also, the results of Palese et al. (2004) indicated that wildfire burning significantly increased soil DOC content. Pardini et al. (2004) reported that the DOC concentration in runoff from cultivated olive trees was four times higher following a wildfire. Furthermore, in a previous study, Zhou et al. (2015) found the mineralization of the soil and the erosion of the humus-rich topsoil layer were significantly enhanced, resulting in a continuous decrease of soil organic matter in young Chinese fir forests. In addition, release of DOM from Chinese fir litter to soil is a limited and slow process, starting when the leaves of Chinese fir trees die (Xue, 1996). Decomposition of the limited Chinese fir litter that accumulates on the ground is slow since its *C*/*N* ratio of up to 134 (Wei et al., 2012) is much larger than the *C*/*N* ratios of Chinese fir soils (about 13.3) (Wan et al., 2015). Therefore, it usually takes about 6 years for 95% degradation of the leaves of Chinese fir to occur (Zhou et al., 2015). This inevitably reduces the rate of decomposition of the litter, lessens the accumulation of organic matter, and slows the release of DOM during the early stage of Chinese fir plantations.

With increasing forest age, soil TN, TP, TK, and NO_3_^–^ concentrations increased significantly (Figures 1B–D,F). These changes are mainly attributable to the development of vegetation, litter nutrient return, interaction between soil and vegetation, biotic fixation, and atmospheric deposition (Knops and Tilman, 2000; Zhang et al., 2010). Litterfall and the return of nutrients are important components of forest biogeochemical and nutrient cycling. It has been reported that litterfall associated with nutrient deposition provided 30–40% of K, >90% of N and P for uptake by plants, and 70–90% of the total nutrient demand of plants (Campos et al., 2017; Zhu et al., 2019). For example, in Chinese fir plantations, annual litterfall production increased with stand age and was 3,295, 3,734, and 4,876 kg ha^–1^ a^–1^ in stands aged 10, 22, and 34 years, respectively (Zhou et al., 2015). Also, the litterfall production nutrient return of N, P, and K input to soil was significantly greater in the old-aged stand (34 years) than in the young-aged stand (10 years). However, in our study, soil NH_4_^+^, AP, and AK concentrations did not increase with stand age (Figures 1G–I). This may be due to most of the P in the litter being converted to organic P due to decomposition by microorganisms (Vincent et al., 2010) and fixation of available P by iron and aluminum oxides (Liu J. et al., 2016). The absence of increased soil available K may be associated with increased undergrowth plant uptake and leaching (Velázquez et al., 2016). Soil pH changes were not obvious with Chinese fir stand development (Supplementary Table 2).

### Characterization of Dissolved Organic Matter Chemodiversity

**FIGURE 2:**
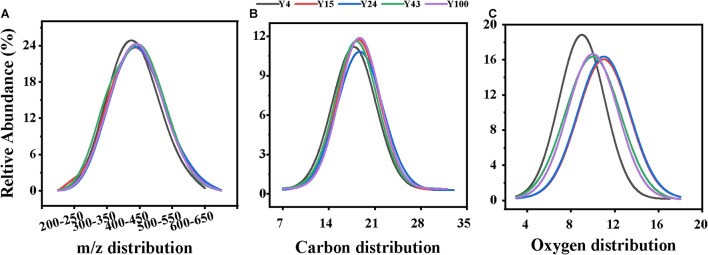
The relative abundance of different properties of soil dissolved organic matter (DOM) molecules in Chinese fir plantation stands of different ages: **(A)** carbon numbers, **(B)** oxygen numbers, and **(C)** *m*/*z* distribution. A Gaussian function was used to fit the curves.

The characteristics of DOM can reveal the unique molecular composition of soils after different durations of Chinese fir plantations. As shown in Table 1 and Figure 2, compared with the 4-year-old Chinese fir plantation, higher soil sample *m*/*z* values occurred in the older stands, which may be due to polymerization reactions, where small molecular substances become macromolecular substances. In contrast to this, the average *H*/*C* value decreased with stand age, indicating an increase in the aromaticity of the DOM. Aligned with the decreased *H*/*C* value, the DBE increased from 8.13 to 9.02, indicating that the degree of molecular unsaturation increased, showing greater recalcitrance with Chinese fir stand development (Thieme et al., 2019). This may be because bacteria can release high-molecular-weight materials with a highly unsaturated and aromatic structure as byproducts (Bai et al., 2017; Roth et al., 2019).

**TABLE 1:** Intensity-weighted average (wa) values and chemodiversity indexes for the molecular composition of soil DOM under different stand ages of Chinese fir plantations.

Stand age, years	*O*/*C*_wa_	*H*/*C*_wa_	DBE_wa_	*m*/*z*_wa_	Shannon index	Pielou index
4	0.55	1.21	8.13	415.84	7.59	0.92
15	0.56	1.16	8.95	428.96	7.60	0.92
24	0.55	1.17	8.91	427.53	7.63	0.93
43	0.56	1.16	8.76	421.38	7.41	0.91
100	0.54	1.14	9.02	423.25	7.52	0.92

**FIGURE 3:**
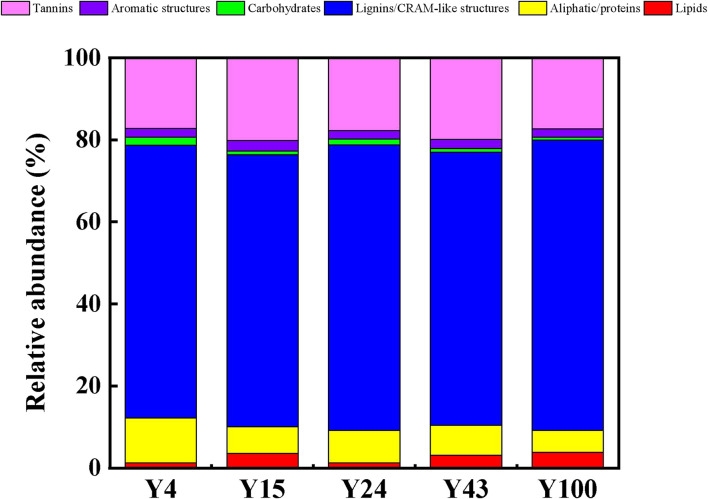
Comparison of the composition of soil dissolved organic matter (DOM) from different stand ages of Chinese fir plantations.

Based on their *O*/*C* and *H*/*C* ratios as well as major subcategories, the identified DOM formulae were visualized in a van Krevelen diagram and were grouped into seven distinct biochemical classes according to previous literature reports (Jones and Willett, 2006; Li et al., 2018) as follows: lipids, aliphatic/proteins, lignins/carboxylic-rich alicyclic molecule (CRAM)-like structures, carbohydrates, unsaturated hydrocarbons, aromatic structures, and tannins. Changes in soil DOM during stand development can be assessed by comparing the relative abundance of major compound classes in each soil. Consistent with the results of Zhang C. et al. (2016) in conifer species (*Picea abies* and *P. sylvestris*), the lignin/CRAM-like structures accounted for the largest proportion of DOM, followed by tannin-like structures (Figure 3). This soil DOM composition is consistent with the chemical composition of Chinese fir litter, which mainly comprise lignin, tannin, and resin (Bai et al., 2017). During decomposition of leaf litter, loss of labile compounds (e.g., carbohydrates and amino acids) occurs, and the dominance of lignin-like structures increases (Bai et al., 2017). In the current study, the relative abundance percentages of lignin/CRAM-like structures and lipid-like compounds were highest in the oldest 100-year-old stands. In contrast, both aliphatic/protein-like and carbohydrate-like compounds decreased with Chinese fir stand development. This is because the carbohydrate-like compounds are degraded more easily, meaning that a higher proportion of the DOM left behind is lignin. For example, Thieme et al. (2019) reported that lignin-like biomolecules accounted for 59% of DOM formulae in litter leachate in coniferous forest, although the proportion was lower (14%) in unmanaged *Fagus sylvatica* L.-dominated plots. In addition, lignin is generally more difficult to degrade by microorganisms, and only a small number of microorganisms (mainly fungi) can produce lignin-degrading enzymes (Tuomela et al., 2000).

The Shannon index of DOM compounds revealed some evidence of a decline in diversity with Chinese fir stand age (Table 1). The microbiological analysis conducted in this study showed that the soil fungal OTU and diversity were lowest during the early stage of Chinese plantation (Supplementary Table 3). Therefore, the release of lignin from litter into the soil requires a comparatively long time, and the lignin-like compounds in DOM are more difficult to degrade by microorganisms, eventually leading to the accumulation of lignin in the subsoil in soluble form. The low bioavailability of lipid-like molecules may be the result of the hydrophobic nature of alkyl carbon compounds, which can prevent access to degrading enzymes (Bai et al., 2017). Zhou et al. (2019) showed that aliphatic/protein-like compounds contain fewer aromatic rings and have a lower oxidation state than lignin-like compounds, which are more susceptible to microbial degradation and are preferentially consumed. It is generally accepted that the initially widely differing chemistry of the leaf litter from distinct plant species converges during decomposition (Strickland et al., 2009; García-Palacios et al., 2016), which is a result of the increasing loss of labile compounds (e.g., carbohydrates and amino acids) and the increasing dominance of lignin (García-Palacios et al., 2016).

**FIGURE 4:**
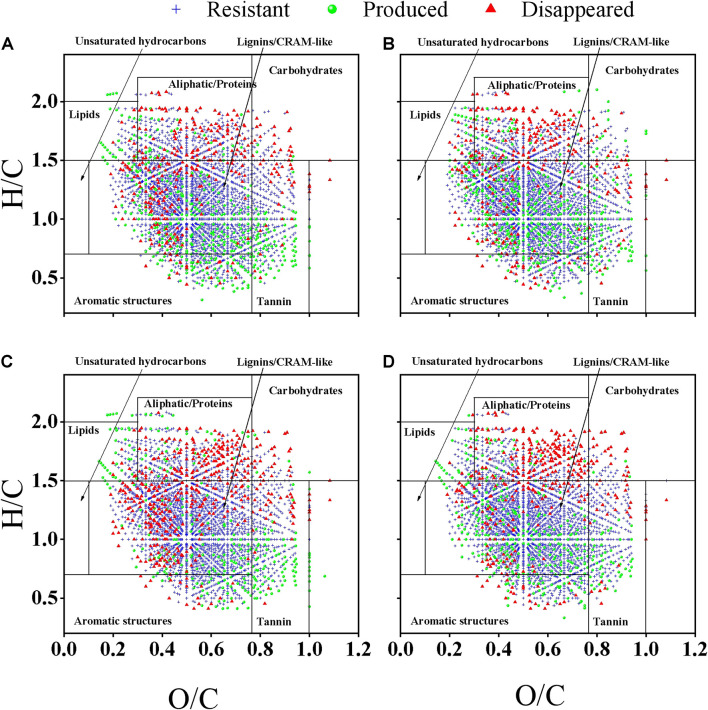
van Krevelen diagrams of the changes in biochemical compound classes of soil DOM between different stand ages of Chinese fir plantation. **(A)** A comparison between 4- and 15-year-old sites. **(B)** A comparison between 4- and 24-year-old sites. **(C)** A comparison between 4- and 43-year-old sites. **(D)** A comparison between 4- and 100-year-old sites. Points in red represent DOM molecules that disappeared with stand development, points in blue represent unchanged DOM molecules, and points in green represent new molecules that appeared during stand development.

In the current study, there was no obvious change in the proportion of tannin-like DOM with forest plantation age, which may indicate that tannins are toxic to microorganisms (Bai et al., 2017). In particular, the formation of tannin-like protein complexes can inhibit microbial processes (such as decomposition) by affecting microbial activity or by changing the microbial community composition (García-Palacios et al., 2016). In contrast, based on analysis of DOM in soil leachate below grassland plots under the same soil type, Roth et al. (2019) suggested that microbial decomposition is the primary driver of soil DOM chemodiversity rather than chemical recalcitrance, with small plant-derived molecules consumed by microbes and transformed into larger microbial-derived molecules. In our study, the content of plant-derived DOM molecules (such as lignins and tannins) remained stable or increased slightly with stand age, while microbially derived DOM compounds (such as aliphatic/proteins) decreased gradually (Figures 3, 4). This difference in soil DOM chemodiversity at our sites may be explained by differences in soil chemistry (discussed in “Soil Nutrient Content of Chinese Fir Plantations of Different Ages” section) and microbial composition between them (see “Characterization of Microbial Community Composition” section).

In the soil of Chinese fir forests, with increasing plantation age, the newly produced DOM is mainly lignin/CRAM-like or tannins with high *O*/*C* ratio and low *H*/*C* ratio (Figure 4). At the same time, part of the DOM components disappeared, in which most of them had low DBE and a low number of C atoms or high DBE and a high number of C atoms, especially aliphatic/proteins and carbohydrates; low *O*/*C* ratio lignins/CRAM-like and aromatic structures; and high *H*/*C* tannin substances (Figure 3 and Supplementary Figures 2, 3). This indicated that the lower *O*/*C* and higher *H*/*C* molecules in soil DOM in Chinese fir plantation were more easily decomposed. Furthermore, most newly formed molecules had high DBE and a high number of C atoms (Supplementary Figure 2) and O atoms (Supplementary Figure 3). The newly formed DOM molecules with high *O*/*C* ratio and low *H*/*C* ratio may be released from the degradation of litter, such as the tannin-like compounds, which are difficult to decompose. This may be because microorganisms are more likely to utilize lower *O*/*C* substances (Lusk and Toor, 2016; Bai et al., 2017; Yuan et al., 2017). Our result is similar to that of Yuan et al. (2017), who reported that biological degradation affected the *O*/*C* ratios of DOM in leachate concentrate by consuming the oxygen-deficient substances and producing oxygen-rich substances.

### Characterization of Microbial Community Composition

**FIGURE 5:**
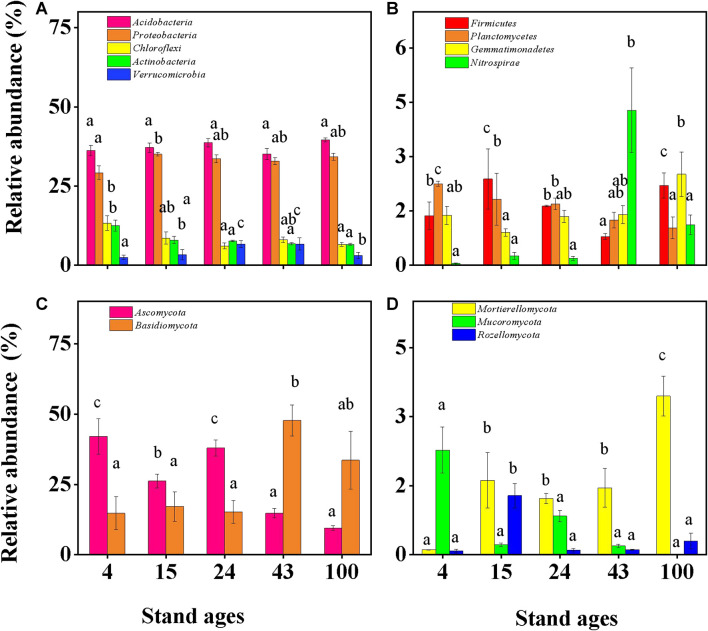
Soil bacteria and fungi with relative abundance above 1% across the Chinese fir planting chronosequence (mean ± SE, *n* = 3). **(A,B)** Relative abundances of components of the bacterial community composition at the phylum level. **(C,D)** Relative abundances of components of the fungal community composition at the phylum level. Bars with different letter(s) are significantly different between stand ages (*p* < 0.05) for each individual phylum.

The structure of the microbial communities differed significantly in Chinese fir forest soils depending on the stage of stand development (Figure 5 and Supplementary Figures 4, 5). The richness and diversity of soil bacteria increased with Chinese fir stand development (Supplementary Table 3) (Cao et al., 2021), which is consistent with the results of Zhang C. et al. (2016). *Acidobacteria* (36.3–39.6%) and *Proteobacteria* (29.2–34.2%) dominated soil bacterial communities in all stand ages (Figure 5A), as previously reported in Chinese fir plantations (Wang et al., 2018). *Acidobacteria* have been reported to be efficient colonizers of acidic terrestrial habitats, such as the pH 4.2–4.9 soils in the study stands, due to their ability to breakdown and use a diverse range of carbohydrates and to hydrolyze various biopolymers (Belova et al., 2018). This study found no significant change in *Acidobacteria* with increasing plantation ages, while the relative abundances of *Chloroflexi*, *Actinobacteria*, and *Planctomycetes* decreased significantly (Figures 5A,B). A significant increase was found in *Proteobacteria* between stand ages 4 and other stand ages. Compared to other forest ages, *Gemmatimonadetes* had the greatest abundance in the 100th year. Furthermore, the *Nitrospirae* increased significantly only in stand age 43 years compared to other stand ages (Figures 5A,B).

The relative abundances of soil bacterial communities at different ages showed different dynamic patterns. Bacterial changes may be due to differences in their metabolic types and their ability to adapt to changes in environmental conditions and nutrient availability. *Acidobacteria* represent one of the most abundant and ubiquitous bacterial phyla found in global soil environments, inhabiting a wide variety of natural environments over a range of temperature, salinity, organic matter, and pH (Jones et al., 2009; Rawat et al., 2012). According to previous reports, the abundance of *Acidobacteria* negatively correlates with soil pH (Kishimoto et al., 1991; Kleinsteuber et al., 2008; Lauber et al., 2009). In the present study, the abundance of *Acidobacteria* did not change significantly with forest age, which was consistent with the stable and acidic soil pH across stand ages (Supplementary Table 2).

It has been hypothesized that elevated soil N should decrease the abundance of oligotrophic taxa, while increasing the abundance of copiotrophic taxa (Fierer et al., 2012; Li et al., 2017; Nie et al., 2018). Results of the current study supported this hypothesis, with decreased oligotrophic taxa (represented by taxonomic groups of *Chloroflexi*) and increased copiotrophic taxa (represented by *Proteobacteria*) (Figure 5A) occurring with increased soil TN as Chinese fir stand age increased (Figure 1B).

Similarly, soil fungal richness and diversity first increased and then decreased across the Chinese fir stand age gradient in the current study (Supplementary Table 3). This result was consistent with previous research in *Pinus tabulaeformis* forests on the Loess Plateau, China (Tian et al., 2017), and suggests that forest age may be a positive controlling factor of the development of fungi in forest soil. As others have reported across ecosystems such as grasslands (Leff et al., 2015), forests (Yu et al., 2013), and pea fields (Xu et al., 2012), the dominant soil fungal phyla in the current study were *Basidiomycota* and *Ascomycota* (Figures 5C,D).

The dominant fungi in Figures 5C,D show different trends depending on the age of the forest. *Ascomycota* were the most abundant fungi during the early stage of Chinese fir plantations (up to 42.1% relative abundance), but with increasing forest age, their relative abundance decreased to 9.5%. Its dominance was gradually replaced by that of *Basidiomycota*, increasing from 14.6% initial relative abundance to 47.7% (Figure 5C). Most of the *Ascomycota* fungi are saprophytic, can degrade soil organic matter, and have a rapid evolution rate. This may be because *Ascomycetes* are more suited for growth in nutrient-poor environments as previously reported that close to 46% of *Ascomycota* can promote lichen formation and can persist and grow in deserts and on mountaintops (Tian et al., 2017). Therefore, in alignment with these properties, *Ascomycetes* were mainly present during the early stage of succession in plant systems. In contrast, *Basidiomycota* (white-rot fungi) have a relatively slow evolution rate and mainly appear during the late stage of succession (Poll et al., 2010). *Basidiomycota* can cause wood decay, have a strong ability to decompose lignocellulose, and are the key decomposers of organic matter in soil.

*Mortierellomycota* are members of *Mucoromyceta*, based on recent fungal taxonomy. They are sporangiferous, generally saprotrophic, have the ability to grow on other fungi, and are found in soil. The relative abundance of *Mortierella* increased with forest stand age (Supplementary Figure 5B). As slow-growing R-strategist fungi, *Mortierella* have been described to mineralize readily available DOM rather than decomposing litter polymers in soil (Suleiman et al., 2019). In contrast, the relative abundance of *Mucoromycota* decreased with forest stand age. *Mucoromycota* is a more derived clade of Zygomycetes and is primarily composed of root endophytes, mycorrhizal fungi, and decomposers of litter, which are crucial for soil nutrient status (Poll et al., 2010), as previous research reported that *Mucoromycota* could improve plant nitrogen use efficiency and total plant yield (Li et al., 2009; Zhang M. et al., 2019). The current study showed that soil NO_3_^–^N concentration was negatively related with *Mucoromycota* abundance (Supplementary Figure 6), which implies that increased N availability in Chinese fir soils decreases the abundance of *Mucoromycota.* Similarly, short- and long-term N fertilization experiments on grassland field plots have demonstrated that increased N availability decreases the abundance of mycorrhizal fungi (Bradley et al., 2006).

### Linkages Between Dissolved Organic Matter Molecular Composition, Chemical Properties, and Soil Microbial Communities

**FIGURE 6:**
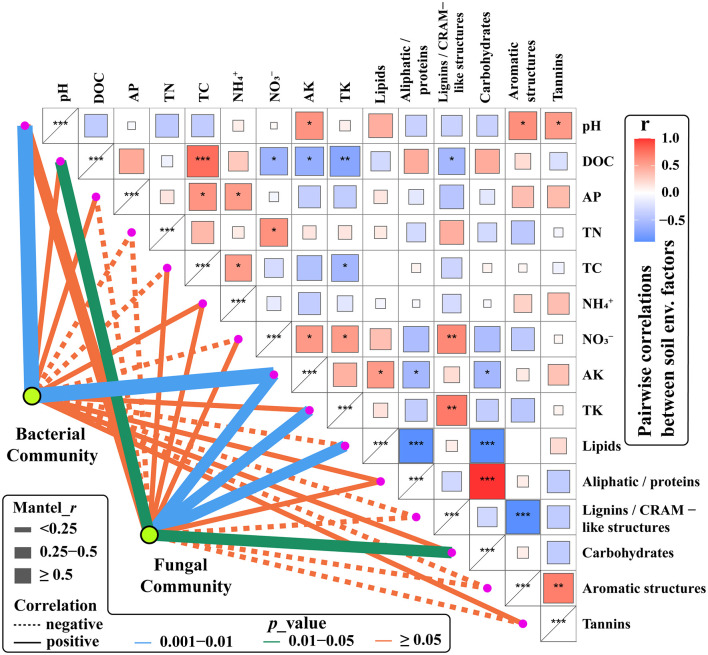
Partial Mantel tests of pairwise comparisons of soil chemical properties and DOM composition with bacterial and fungal communities. Line width is proportional to the Mantel *r* values, line color denotes the significance level, and lien formatting indicates the correlation sign (solid line is a positive correlation and dashed lines are a negative correlation). ***: Significantly correlated at 0.001 level; **: Significantly correlated at 0.01 level; *: Significantly correlated at 0.05 level.

To determine the soil environmental driving factors in bacterial and fungal communities, we correlated bacterial and fungal communities (OTUs) with environmental factors (DOM composition and chemical properties) (Figure 6). Soil pH and AK were strongly positively correlated with the bacterial community. Similarly, soil pH and AK have been identified as key drivers of the bacterial community in alpine system, by affecting the adaptation and selection process of particular bacterial phylogenic groups (Wang et al., 2020). There were moderate positive correlations between fungal community and AK, TK, DOC, lipids, and carbohydrates, similar to the findings of Siles and Margesin (2016).

The lignin/CRAM-like structures in DOM were positively correlated with *Proteobacteria* and *Mortierellomycota* relative abundances. However, they are negatively correlated with *Chloroflexi* and *Mucoromycota* (Supplementary Figure 7). It has been reported that *Proteobacteria*, including *Pseudomonas*, *Burkholderia*, and *Enterobacter*, can degrade lignin from litter (Brink et al., 2019) to produce more soluble lignin/CRAM compounds. The bacterial degradation of lignin mainly occurs at the primary metabolic stage, and lignin-degrading enzymes are synthesized during both the logarithmic growth phase and the stationary phase (Brink et al., 2019). Bacterial degradation mainly converts lignin into a water-soluble polymer to a certain extent, which is rarely a complete mineralization, and, typically, bacteria are slow to degrade lignin (Tuomela et al., 2000). However, in most cases, bacteria exert an indirect effect on lignin degradation, i.e., their synergy with fungi accelerates lignin degradation. Fungal degradation of lignin occurs mainly during the secondary metabolic stage and relies on enzymatic catalysis to produce chemically unstable lignin-free reactive intermediates, followed by a series of spontaneous degradation reactions (Tuomela et al., 2000). The operation of these processes is indicated in the present study by the positive correlation of *Mortierella* (phylum *Mortierellomycota*) relative abundance with lignin/CRAM-like DOM and lipid-like DOM, but its negative correlation with carbohydrate-like DOM (Supplementary Figure 8). This interpretation is supported by the results of Zeng et al. (2013), who reported that *Mortierella* are able to use a wide range of carbohydrates to break down lignocellulose (cellulose and lignin hemicellulose, cellulose, and lignin) from wheat straw to produce lipids. Supplementary Figure 7 also shows that *Basidiomycota* had a positive correlation with tannin-like DOM. As Prigione et al. (2018) report, the suitability to grow on matrix rich in tannins and on industrial tannin preparations is usually a recognized characteristic of some species of fungi such as *Basidiomycota*. These microbes can tolerate the toxicity of tannins owing to the production of enzymes that degrade or transform these substrates, largely through oxidation and hydrolysis, and some can utilize tannins as the single carbon source (Prigione et al., 2018).

**FIGURE 7:**
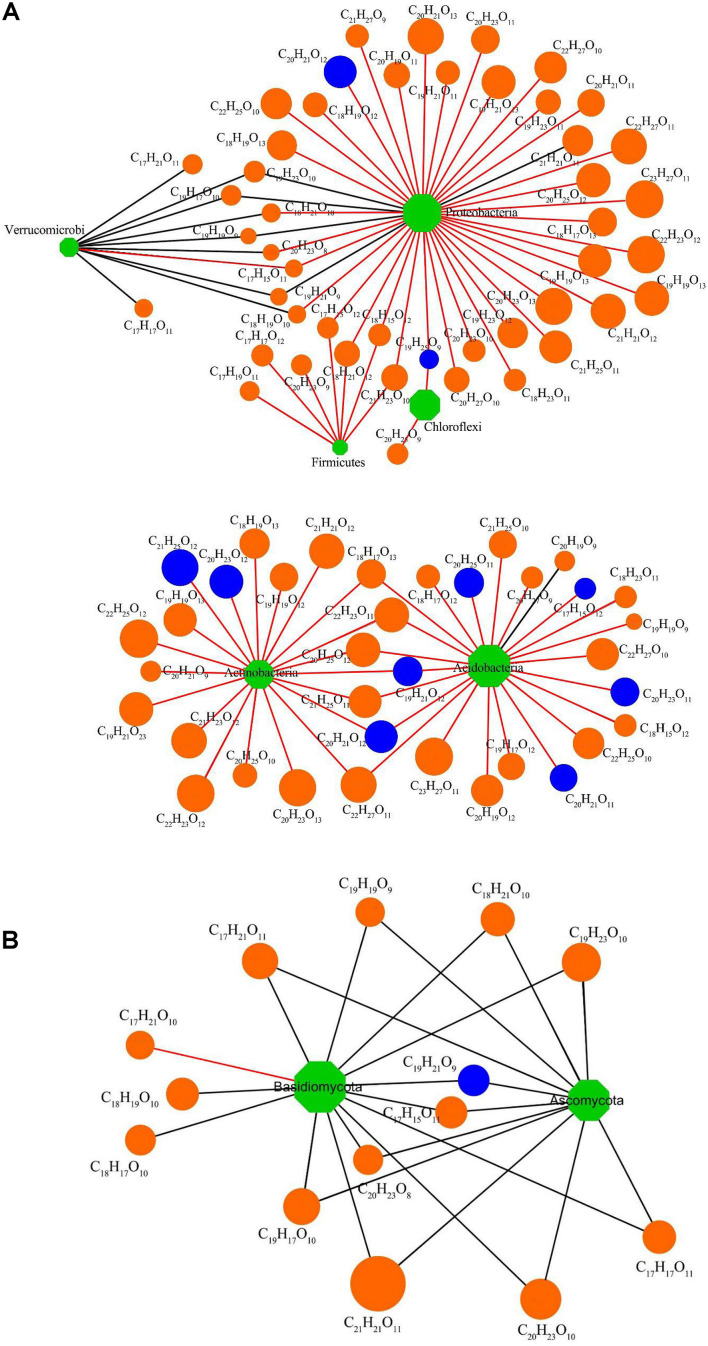
**(A)** Interaction network analysis of the 100 most abundant bacterial operational taxonomic units (OTUs) and the 100 most abundant DOM molecules with significant correlation (*p* < 0.01). **(B)** Interaction network analysis of the 50 most abundant fungal OTUs and the 100 most abundant DOM molecules with significant correlation (*p* < 0.01). Circles are DOM molecules [orange, lignins/carboxylic-rich alicyclic molecules (CRAM) and blue, tannins]. Rhombuses are bacterial and fungal OTUs (green). DOM molecules’ relative abundances are proportional to node size. Positive correlations are indicated using red lines and negative correlations using black lines.

The interconnections between the DOM chemodiversity and the soil bacterial community were examined by a co-occurrence network analysis using the 100 most abundant DOM molecules and the 100 bacterial OTUs with the highest relative abundance. The results showed different links between DOM molecules and soil bacteria (Figure 7A). Three hundred seventy correlations were found, of which 41 linked 27 DOM molecules with 22 OTUs at *p* < 0.01. These 22 OTUs were dominant members at the taxonomic level of the bacterial phylum. The bioavailable DOM is now considered as a continuum with labile, semi-labile, and refractory pools that are identified by uninterrupted turnover lives from a few minutes to multiple millennia (Peri et al., 2006; Liu et al., 2019). These fractions influence the biogeochemistry related to bacterial community succession, transmigration, and energy and carbon cycles of ecosystems (Liu et al., 2019). The 22 OTUs belonged to *Acidobacteria* (four OTUs), *Proteobacteria* (12 OTUs), *Verrucomicrobia* (one OTU), *Actinobacteria* (three OTUs), *Firmicutes* (one OTU), and *Chloroflexi* (one OTU). Among these, *Acidobacteria*, *Chloroflexi*, and *Firmicutes* were positively correlated with some DOM molecules, while *Proteobacteria* was positively correlated with most DOM molecules and negatively correlated with a few DOM molecules. In contrast, *Verrucomicrobia* was negatively correlated with most DOM molecules (Figure 7A). The DOM molecules related to bacteria mainly belong to lignin/CRAM-like structures. A few were tannin-like, and one was lipid-like, indicating that lignin/CRAM-like structures and tannin-like structures became more stable in soil with planting duration. Studies have shown that lignin/CRAM-like structures contain more aromatic rings and higher oxidation states than lipids and aliphatic/proteins; thus, they were more recalcitrant for bacterial decomposition (Boye et al., 2017). These results highlight that specific bacteria can only produce or decompose specific DOM molecules. Moreover, different components of DOM change at different speeds (Follett et al., 2014).

The 100 most abundant DOM molecules and the fungal 50 OTUs with the highest relative abundance were also selected for network analysis. After correlation analysis, 127 correlations were found, of which 25 linked 16 DOM molecules and 10 fungal OTUs at *p* < 0.01 (Figure 7B). These significantly correlated OTUs were dominant members of *Basidiomycota* (two OTUs) and *Ascomycota* (eight OTUs). A significant negative correlation was found between *Ascomycota* and all DOM molecules, while *Basidiomycota* was negatively correlated with most DOM molecules but positively correlated with one kind of DOM molecules. DOM molecules related to fungi mainly belonged to lignin/CRAM-like structures, followed by tannins and one aliphatic/protein structure (Figure 7B). Since positive correlations between DOM molecules and microorganisms have been considered indicative of biodegradation (Zhou et al., 2019b), the negative correlations here may be interpreted as evidence that *Basidiomycota* and *Ascomycota* have limited ability to degrade lignin/CRAM-like and tannin molecules in soils underlying Chinese fir plantations.

## Conclusion

This study demonstrates that the soil nutrient content increased significantly with Chinese fir plantation age and tended to be stable during the later stage of stand development from 4 to 100 years. Soil bacterial richness and diversity increased significantly with stand age, while soil fungal diversity tended to increase during the young plantation stage, and then plateaued during the later stages of stand development. High *H*/*C* DOM molecules in DOM such as lipids and aliphatic/proteins degraded preferentially, while low *H*/*C* DOM such as lignin/CRAM-like structures and tannins showed recalcitrance in the process of stand development due to the different preferences of soil microorganisms for the decomposition and utilization of organic matter. More importantly, the results confirmed that microbes play an important role in the transformation of plant-derived organic matter. Significant correlations were found between components of the soil microbial community and DOM composition, which indicates a complex connectivity and strong interaction between the soil microbial community and DOM molecules over the process of Chinese fir stand development. The results of this study improve the general understanding of the interaction between soil DOM and microorganisms and the fate of DOM during monoculture-planted forest stand development. We concluded that DOM is not all inherently recalcitrant but instead is persistent in soil as a consequence of simultaneous transformation, consumption, and formation with extended stand ages of Chinese fir plantations. The conclusions shed new light on the evaluation of the functional recovery of degraded ecosystems, following Chinese fir stand development. In this study, only the DOM component of soil organic matter was considered, but it should be noted that the non-soluble soil organic matter will also influence the microbial community composition and activity. The results of this study can indicate management practices to forest managers and policymakers to preserve soil quality. Specifically, extending the time between planting and harvesting of Chinese fir plantations will be beneficial in enriching the chemical diversity of DOM, which will facilitate microbial reproduction, thus contributing to the improvement of soil ecosystem function.

## Data Availability Statement

The datasets presented in this study can be found in online repositories. The names of the repository/repositories and accession number(s) can be found below: NCBI (accession: PRJNA758192).

## Author Contributions

YL: formal analysis, investigation, validation, data curation, and writing-original draft. KH: supervision and writing – review and editing. SW: validation, writing, formal analysis, and data curation. SC: investigation, validation, resources, and software. CZ: conceptualization, visualization, resources, and supervision. All authors contributed to the article and approved the submitted version.

## Conflict of Interest

The authors declare that the research was conducted in the absence of any commercial or financial relationships that could be construed as a potential conflict of interest.

## Publisher’s Note

All claims expressed in this article are solely those of the authors and do not necessarily represent those of their affiliated organizations, or those of the publisher, the editors and the reviewers. Any product that may be evaluated in this article, or claim that may be made by its manufacturer, is not guaranteed or endorsed by the publisher.

## Footnotes

1
http://www.cloudtutu.com
