# Corrigendum: Chemodiversity of soil dissolved organic matter and its association with soil microbial communities along a chronosequence of Chinese fir monoculture plantations

**DOI:** 10.3389/fmicb.2022.980161

**Published:** 2022-08-08

**Authors:** Ying Li, Kate Heal, Shuzhen Wang, Sheng Cao, Chuifan Zhou

**Affiliations:** ^1^University Engineering Research Center of Sustainable Plantation Management, Forestry College, Fujian Agriculture and Forestry University, Fuzhou, China; ^2^Institute of Quality Standards and Testing Technology for Agro-Products, Fujian Academy of Agricultural Sciences, Fuzhou, China; ^3^School of GeoSciences, The University of Edinburgh, Edinburgh, United Kingdom

**Keywords:** DOM, soil quality, bacteria, fungi, Chinese fir, chemodiversity

In the published article the reference “Cao, S., Pan, F., Lin, G. G., Zhang, Y. L., Zhou, C. F., and Liu, B. (2021). Changes of soil bacterial structure and soil enzyme activity in Chinese fir forest of different ages. *Acta Ecologica Sinica*. 41, 1846–1856.” was not cited.

The citation has now been inserted in **Materials and Methods**, “*Soil Sampling and Preparation*,” first paragraph and should read: “In 2018, three replicate stands were selected in Chinese fir plantations of five different ages (4, 15, 24, 43, and 100 years, the soil background information shown in Fig. 1 and images shown in Supplementary Figure 1) for the study (Cao et al., 2021)”.

The citation has also been inserted in **Results and Discussion**, “Characterization of Microbial Community Composition” first paragraph and should read: “The structure of the microbial communities differed significantly in Chinese fir forest soils depending on the stage of stand development (Figure 5 and Supplementary Figures 4, 5). The richness and diversity of soil bacteria increased with Chinese fir stand development (Supplementary Table 3) (Cao et al., 2021), which is consistent with the results of Zhang C. et al. (2016).

The authors apologize for this error and state that this does not change the scientific conclusions of the article in any way. The original article has been updated.

## Publisher's note

All claims expressed in this article are solely those of the authors and do not necessarily represent those of their affiliated organizations, or those of the publisher, the editors and the reviewers. Any product that may be evaluated in this article, or claim that may be made by its manufacturer, is not guaranteed or endorsed by the publisher.
