# Supplementary material for: Chemodiversity of Soil Dissolved Organic Matter and Its Association With Soil Microbial Communities Along a Chronosequence of Chinese Fir Monoculture Plantations
Source: Front Microbiol. 2021 Oct 21;12:729344. doi: 10.3389/fmicb.2021.729344 (PMC8566896; doi:10.3389/fmicb.2021.729344)
Supplement: Supplementary file 1 [file Data_Sheet_1.docx]

Supplementary Material for:

**Chemodiversity of soil dissolved organic matter and its association with soil microbial communities along a chronosequence of Chinese fir monoculture plantations**

Ying Li^1,2^, Kate Heal^3^, Shuzhen Wang^1^, Sheng Cao^1^, Chuifan Zhou^1*^

^1 University Engineering Research Center of Sustainable Plantation Management, Forestry College, Fujian Agriculture and Forestry University, Fuzhou, China,^

^2 Institute of Quality Standards and Testing Technology for Agro-Products, Fujian Academy of Agricultural Sciences, Fuzhou, China,^

^3 School of GeoSciences, The University of Edinburgh, Edinburgh, United Kingdom^

*Corresponding author

E-mail: zhouchuifan@163.com

Number of pages: 13

Number of figures: 8

Number of tables: 3

**Table S1.** Key characteristics of sampling plots in different ages of Chinese fir plantations. Tree height is the mean tree height for Chinese fir trees. (Vegetation coverage: coverage of all vegetation species in Chinese fir forests )

| **Stand age (years)** | **Elevation**  **(m)** | **Slope**  **(°)** | **Tree height**  **(m)** | **All Vegetation**  **coverage (%)** | **Main understory vegetation species** |
| --- | --- | --- | --- | --- | --- |
| 4 | 215-245 | 28 | 4.1 | 50 | *Dicranopteris dichotoma* (Thunb.) Berhn.  *Lophatherum gracile* Brongn.  *Stenoloma chusanum* Ching.  *Woodwardia japonica* (L. f.) Sm.  *Cratoxylum cochinchinense* (Lour.) Bl.  *Cyrtococcum patens* (L.) A.Camus  *Argyreia seguinii* (Levl.) Van. ex Levl.  *Rhus chinensis* Miller. |
| 15 | 206-248 | 30 | 11.3 | 70 | *Dicranopteris dichotoma* (Thunb.) Berhn.  *Gahnia tristis* Nees  *Melastoma dodecandrum* Lour.  *Indocalamus tessellatus* (Munro) Keng f.  *Toxicodendron succedaneum* (L.) O.Kuntze  *Daphniphyllum oldhami* (Hemsl.) Rosenth.  *Rhus chinensis* Miller |
| 24 | 219-235 | 29 | 16.8 | 80 | *Dicranopteris dichotoma* (Thunb.) Berhn.  *Indocalamus tessellatus* (Munro) Keng f.  *Gahnia tristis* Nees  *Castanopsis sclerophylla* (Lindl.) Schott  *Alniphyllum fortune* (Hemsl.) Makino |
| 43 | 216-238 | 28 | 26.7 | 90 | *Dicranopteris dichotoma* (Thunb.) Berhn.  *Miscanthus floridulus* (Labill.) Warb. ex K.Schum. & Lauterb.  *Stenoloma chusanum* Ching.  *Oplismenus compositus* (L.) Beauv.  *Heteropanax brevipedicellatus* H.L.Li  *Choerospondias axillaris* (Roxb.) Burtt et Hill  A*lniphyllum fortune* (Hemsl.) Makino  *Smilax arisanensis* Hayata  *Cyclobalanopsis glauca* (Thunb.) Oerst. |
| 100 | 212-240 | 27 | 34.5 | 95 | *Dicranopteris dichotoma* (Thunb.) Berhn.  *Woodwardia japonica* (L. f.) Sm.  *Alpinia chinensis* (Retz.) Rosc  *Ficus hirta* Vahl  *Cyclobalanopsis glauca* (Thunb.) Oerst.  *Smilax arisanensis* Hayata  *Calamus tetradactylus* Hance |

**Table S2.** Soil pH (mean ± SE, n=3 replicate stands) in soils under different stand ages of Chinese fir plantations. Values with the same letter(s) are not significantly different between stand ages (*p* < 0.05).

| **Stand age, years** | **4** | **15** | **24** | **43** | **100** |
| --- | --- | --- | --- | --- | --- |
| pH | 4.23 ± 0.12a | 4.67 ± 0.08a | 4.45 ± 0.10a | 4.93 ± 0.09a | 4.66 ± 0.02a |

**Table S3.** Number of bacterial and fungal operational taxonomic units (OTUs) and measures of α-diversity in soils under different stand ages of Chinese fir plantations. Values are means ± SE of three replicate samples. Different letter (s) within the same row indicate significant difference between stands of different ages (p < 0.05).

| **Type of microorganism** | **Stand age, years** | **OTU** | **ACE** | **Chao1** | **Shannon** |
| --- | --- | --- | --- | --- | --- |
| Bacterial community | 4 | 908±35b | 953±39b | 966±39b | 5.4±0.022b |
|  | 15 | 1047±37a | 1067±13a | 1077±13a | 5.7±0.041a |
|  | 24 | 1007±25a | 1028±24a | 1038±24b | 5.6±0.044b |
|  | 43 | 1013±29a | 1055±16a | 1058±18a | 5.8±0.052a |
|  | 100 | 1056±10a | 1087±5a | 1102±2a | 5.8±0.067a |
| Fungal community | 4 | 400±44b | 420±42b | 422±45b | 3.8±0.15bc |
|  | 15 | 590±33a | 625±35a | 638±41a | 4.2±0.25b |
|  | 24 | 651±25a | 682±34a | 695±40a | 4.7±0.08a |
|  | 43 | 540±17a | 593±14a | 605±16a | 3.7±0.08bc |
|  | 100 | 531±42a | 586±40a | 598±40a | 3.4±0.18c |


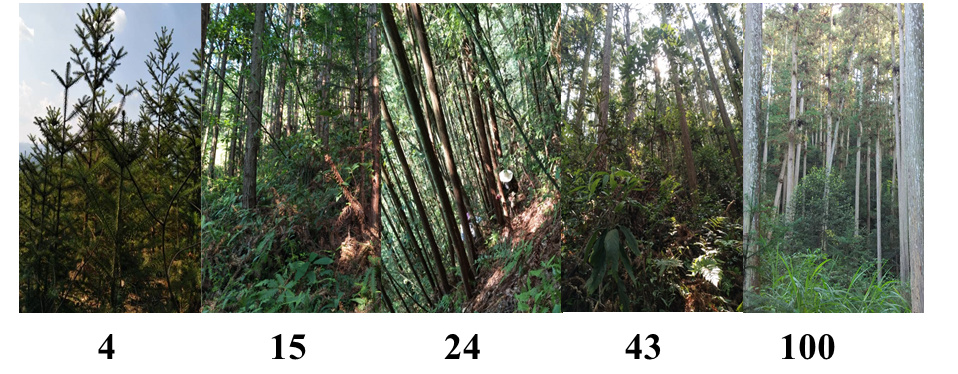


**Fig. S1** Images of the sampling sites with different stand ages of Chinese fir plantations. Stand age in years is shown below each image.

**Fig. S2** Plots of DBE versus carbon number for removed, resistant, and produced soil samples from different stand ages of Chinese fir plantations sites; a: comparison between 4-year old and 15-year old site, b: comparison between 4-year old and 24-year old site, c: comparison between 4-year old and 43-year old site, and d: comparison between 4-year old and 100-year old site. Points in red represent DOM molecules that disappeared with stand development, points in blue represent unchanged DOM molecules, and points in green represent new molecules that appeared between Chinese fir plantations of different ages.

**Fig. S3** Plots of DBE versus oxygen number for removed, resistant, and produced soil samples from different stand ages of Chinese fir plantations sites; a: comparison between 4-year old and 15-year old site, b: comparison between 4-year old and 24-year old site, c: comparison between 4-year old and 43-year old site, and d: comparison between 4-year old and 100-year old site. Points in red represent DOM molecules that disappeared with stand development, points in blue represent unchanged DOM molecules, and points in green represent new molecules that appeared between Chinese fir plantations of different ages.


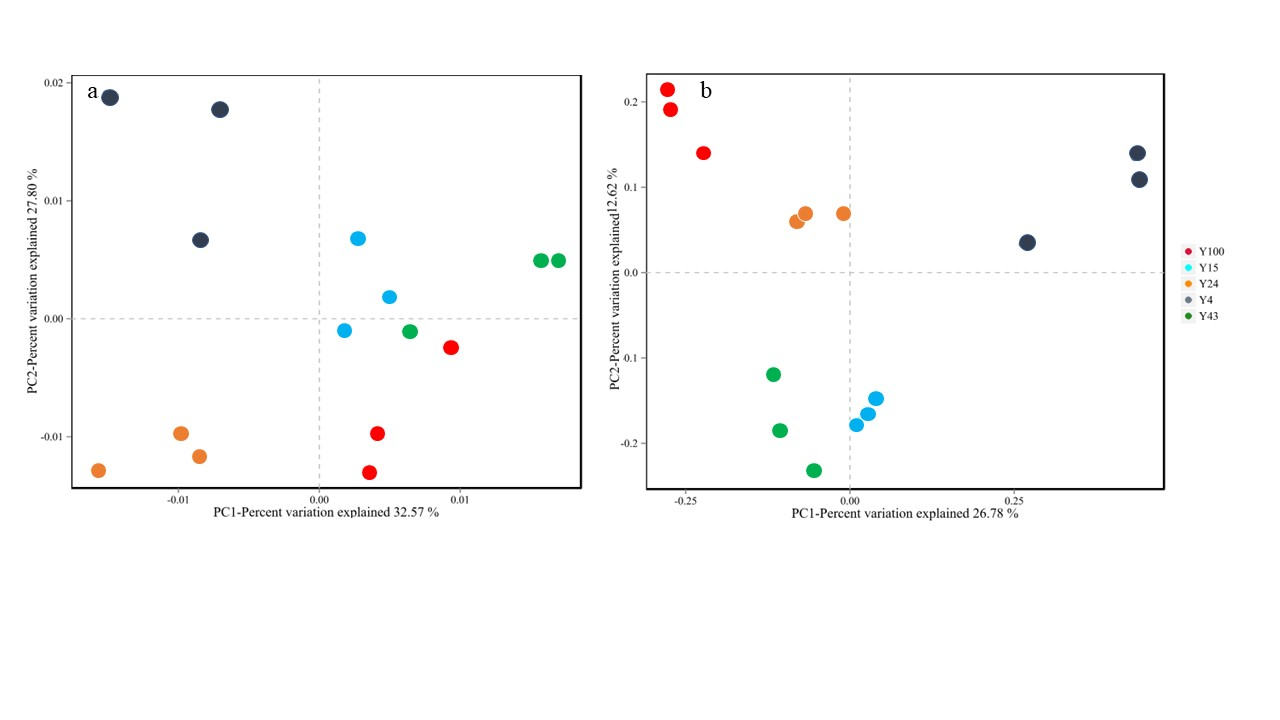


**Fig. S4** Principal component analysis of soil bacterial (a) and fungal (b) communities in different stand ages of Chinese fir plantations.


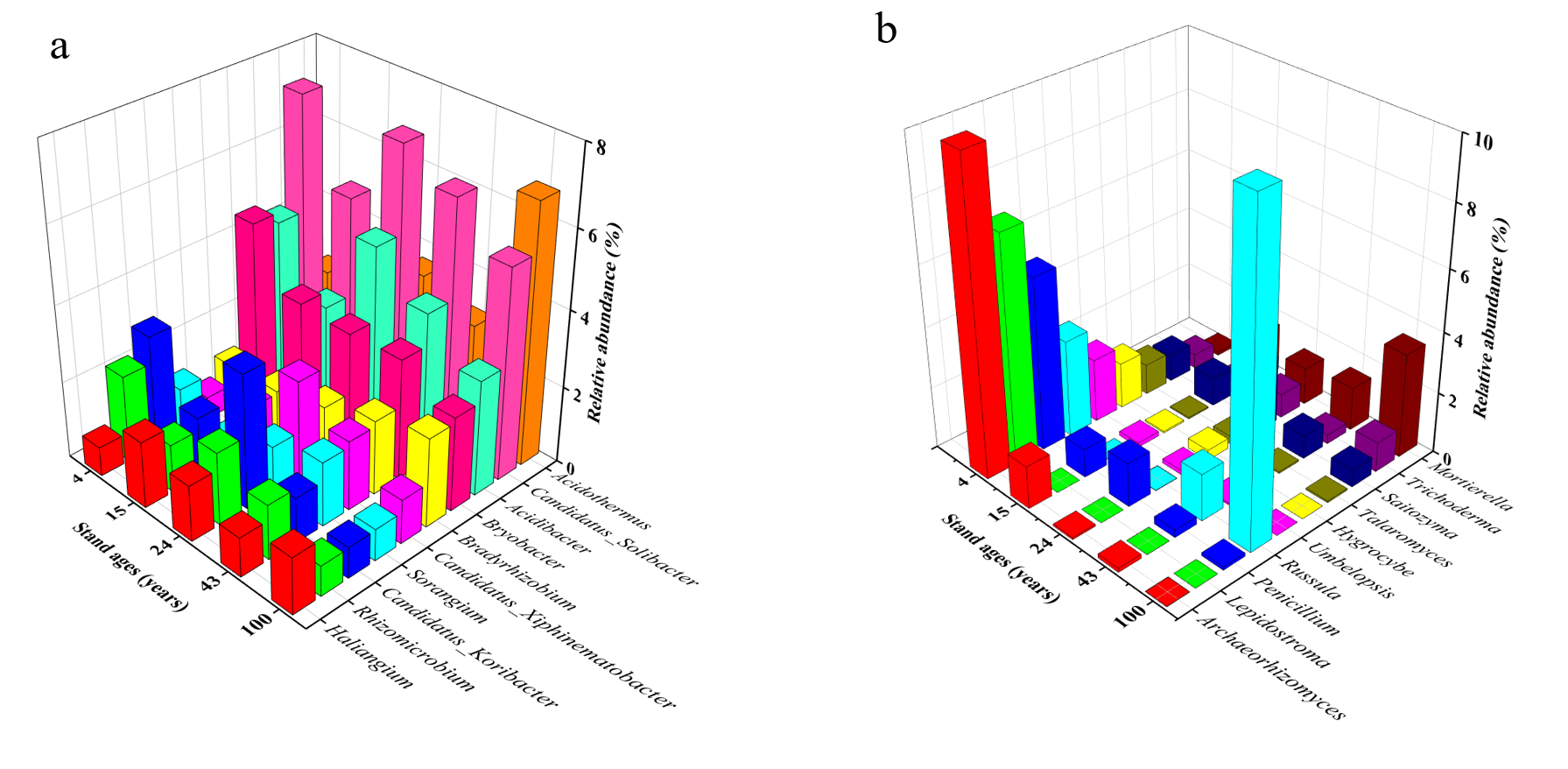


**Fig. S5** Soil bacteria and fungi with relative abundance exceeding 1% (top 10) across a Chinese fir plantation chronosequence. (a) Relative abundances of bacterial community composition components at the genus level. (b) Relative abundances of fungal community composition components at the genus level.

**
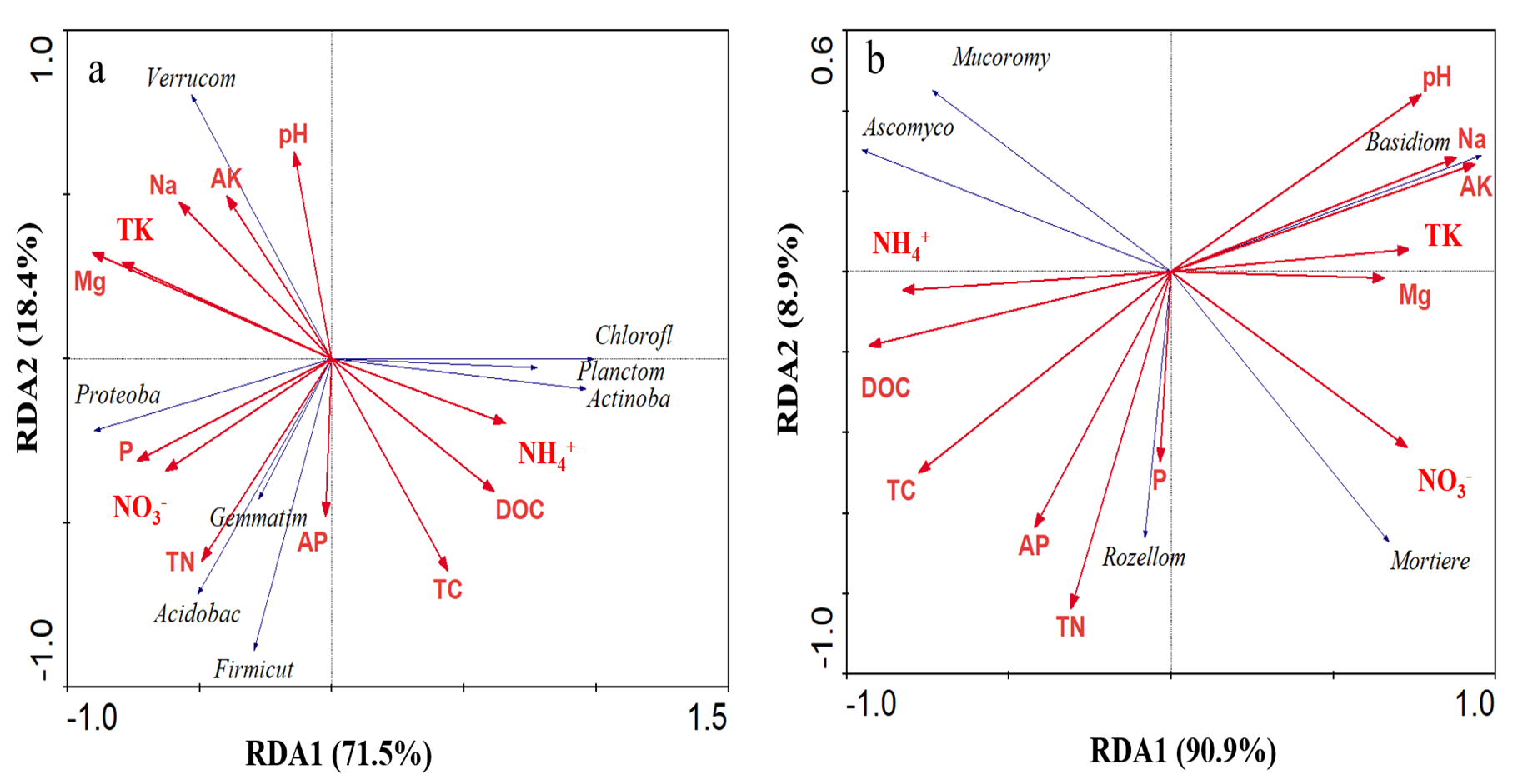
**

**Fig. S6** Redundancy analysis (RDA) ordination plots of the relative abundance of bacterial phylum (a) and fungal phylum (b) exceeding 1% and soil chemical properties across Chinese fir stands of different ages. Bacteria and fungi are represented by blue arrows and soil chemical properties by red arrows. TC: total carbon; TN: total nitrogen; DOC: dissolved organic carbon; NO_3_^-^: Nitrate; NH_4_^+^: Ammonium. Bacteria phylum labels are: *Acidobacteria* (*Acidobac*), *Proteobacteria* (*Proteoba*), *Chloroflexi* (*Chlorofl*), *Actinobacteria* (*Actinoba*), *Verrucomicrobia* (*Verrucom*), *Firmicutes* (*Firmicu*), *Planctomycetes* (*Planctom*), Gemmatimonadetes (*Gemmati*). Fungi phyllum labels are*: Ascomycota* (*Ascomyco*), *Basidiomycota* (*Basidiom*), *Mortierellomycota* (*Mortiere*), *Mucoromycota* (*Mucoromy*), *Rozellomycota* (*Rozellom*).


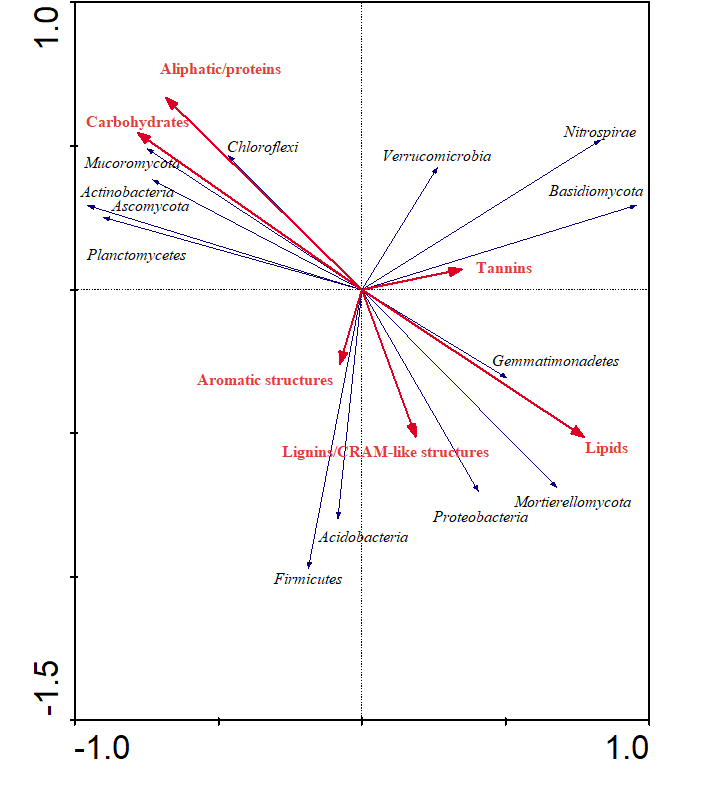


**Fig. S7** Redundancy analysis (RDA) ordination plot of DOM composition (relative abundance) and of bacterial and fungal components (exceeding 1% relative abundance) in soils of Chinese fir plantations of different ages. Bacterial community composition at the phylum level comprises *Acidobacteria*, *Proteobacteria*, *Chloroflexi*, *Actinobacteria* and *Verrucomicrobia*. Fungal community composition at the phylum level comprises *Ascomycota*, *Basidiomycota*, *Mortierellomycota* and *Mucoromycota.*


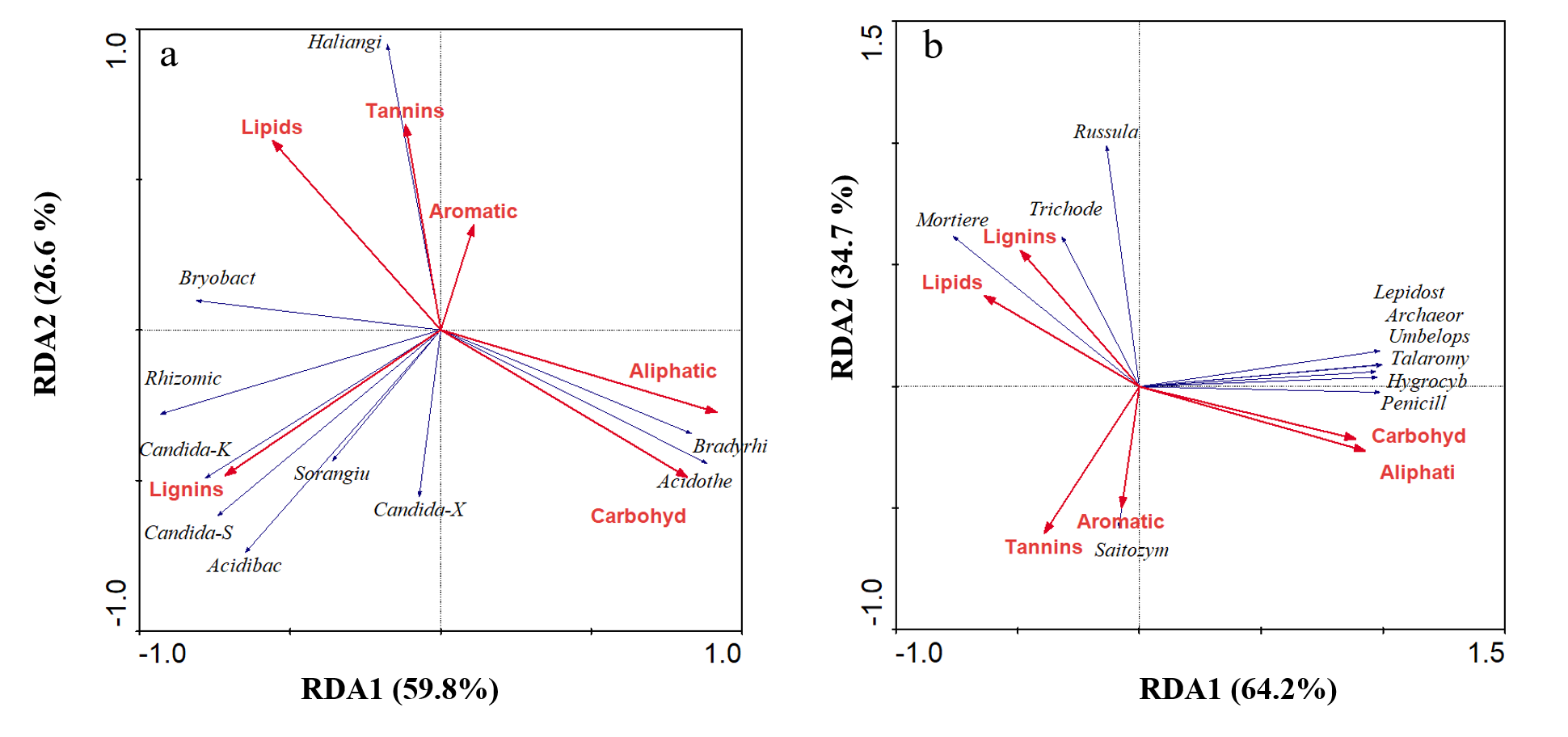


**Fig. S8** Redundancy analysis (RDA) ordination plots based on the relative abundance of bacterial genus (a), fungi genus (b), exceeding 1% and soil DOM composition across Chinese fir stands of different ages. Bacteria and fungi are represented by blue arrows and DOM relative abundance by red arrows. Lignins refers to lignin/CRAM-like DOM, Lipids refers to Lipids-like DOM, Aliphati refers to aliphatic/proteins-like DOM, Carbohyd refers to carbohydrates-like DOM, Aromatic refers to aromatic structure-like DOM, and tannins refers to tannin-like DOM. Bacteria genus labels are: *Candidatus_Solibacter* (*Candida-S*), *Acidibacter* (*Acidibac*), *Acidothermus* (*Acidothe*), *Bryobacter* (*Bryobact*), *Candidatus_Koribacter* (*Candida-K*), *Bradyrhizobium* (*Bradyrhi*), *Rhizomicrobium* (*Rhizomic*), *Candidatus_Xiphinematobacter* (*Candida-C*), *Sorangium* (*Sorangiu*), *Haliangium* (*Haliangi*). Fungi genus labels are: *Archaeorhizomyces* (*Archaeor*), *Lepidostroma* (*Lepidost*), *Penicillium* (*Penicill*), *Russula*, *Umbelopsis* (*Umbelops*), *Hygrocybe* (*Hygrocyb*), *Talaromyces* (*Talaromy*), *Saitozyma* (*Saitozym*), *Trichoderma* (*Trichode*), *Mortierella* (*Mortiere*).
